# Profiling of cellular proteins in porcine reproductive and respiratory syndrome virus virions by proteomics analysis

**DOI:** 10.1186/1743-422X-7-242

**Published:** 2010-09-18

**Authors:** Chengwen Zhang, Chunyi Xue, Yan Li, Qingming Kong, Xiangpeng Ren, Xiaoming Li, Dingming Shu, Yingzuo Bi, Yongchang Cao

**Affiliations:** 1 State Key Laboratory of Biocontrol, School of Life Sciences, Sun Yat-sen University, Guangzhou, 510006, China; 2 State Key Laboratory of Livestock and Poultry Breeding, Institute of Animal Science, Guangdong Academy of Agricultural Sciences, Guangzhou 510640, China; 3 College of Animal Science, South China Agricultural University, Guangzhou, 510642, China

## Abstract

**Background:**

Porcine reproductive and respiratory syndrome virus (PRRSV) is an enveloped virus, bearing severe economic consequences to the swine industry worldwide. Previous studies on enveloped viruses have shown that many incorporated cellular proteins associated with the virion's membranes that might play important roles in viral infectivity. In this study, we sought to proteomically profile the cellular proteins incorporated into or associated with the virions of a highly virulent PRRSV strain GDBY1, and to provide foundation for further investigations on the roles of incorporated/associated cellular proteins on PRRSV's infectivity.

**Results:**

In our experiment, sixty one cellular proteins were identified in highly purified PRRSV virions by two-dimensional gel electrophoresis coupled with mass spectrometric approaches. The identified cellular proteins could be grouped into eight functional categories including cytoskeletal proteins, chaperones, macromolecular biosynthesis proteins, metabolism-associated proteins, calcium-dependent membrane-binding proteins and other functional proteins. Among the identified proteins, four have not yet been reported in other studied envelope viruses, namely, guanine nucleotide-binding proteins, tyrosine 3-monooxygenase/tryptophan 5-monooxygenase, peroxiredoxin 1 and galectin-1 protein. The presence of five selected cellular proteins (i.e., β-actin, Tubulin, Annexin A2, heat shock protein Hsp27, and calcium binding proteins S100) in the highly purified PRRSV virions was validated by Western blot and immunogold labeling assays.

**Conclusions:**

Taken together, the present study has demonstrated the incorporation of cellular proteins in PRRSV virions, which provides valuable information for the further investigations for the effects of individual cellular proteins on the viral replication, assembly, and pathogenesis.

## Background

Porcine reproductive and respiratory syndrome (PRRS) is an economically important disease of swine throughout the world, characterized by severe reproductive problem with late term abortions in sows and severe respiratory ailment leading to increased mortality in young pigs [1,2]. The disease was first reported in the United States in 1987 and subsequently in Europe in 1991, reaching Southeast Asia and Japan in 1995 [3,4]. The disease is now pandemic in many swine-producing countries and has become one of the most serious threats to intensive swine industry. In June 2006, the outbreak of "high fever" in China, caused by highly pathogenic PRRSV infection, spread to more than 10 provinces and took a huge toll in swine industry [5].

Porcine reproductive and respiratory syndrome virus (PRRSV), the causative agent of PRRS, is an enveloped, non-segmented, single positive-stranded virus belonging to the family Arteriviridae in the order Nidovirales [6]. PRRSV produces seven structrual proteins, namely, glycoprotein 2a (GP2a), non-glycosylated protein 2b (or E), GP3, GP4, GP5, the matrix protein (M), and the nucleocapsid protein (N), respectively [7-9]. According to the studies of the closely related equine arteritis virus (EAV), the ORF1a and ORF1b synthesized replicase polyprotein, predicted to be proteolytically cleaved into fourteen nonstructural proteins (NSPs) [10-13].

Numerous host proteins have been identified that incorporate into the membranes or inside the envelopes of the virions during their budding from the host cells, but the role and importance of these host cellular proteins in virus infection are not fully understood [14-16]. Extensive proteomic analysis has been performed on human cytomegalovirus (HCMV) virions, human immunodeficiency virus (HIV), emiliania huxleyi virus 86 (EhV-86) virions, kaposi's sarcoma-associated herpesvirus (KSHV) and influenza virus, that shows the presence of lots of cellular proteins [17-21]. Virion-associated host proteins could be grouped into several functional categories, such as cytoskeletal proteins, annexins, glycolytic enzymes and tetraspanins [20]. TSG101 protein is critical for HIV budding [22]. APOBEC3F exerts its antiviral effect by means of blocking HIV replication [23,24]. Cyclophilin A which impairs the early stage of the viral replication is essential for HIV type 1 virion infectivity [25-27]. Cofliln, Tubulin, heat shock protein (Hsp) 90 and Hsp70 were also detected in Epstein-Barr virus (EBV) [28], while β-actin was identified to interact with infectious bronchitis virus M protein, subsequently confirms to play important roles in virion assembly and budding [29].

However, the identities of the cellular proteins incorporated in PRRSV virions have not been investigated. We infected African green monkey kidney epithelial cell line (Marc-145) with PRRSV and purified the virions by Cesium chloride (CsCl) gradients centrifugation coupled with sucrose gradients centrifugation. The highly purified PRRSV virions were analyzed by two-dimensional gel electrophoresis (2-DE) coupled with mass spectrometric approaches, that identified sixty one different cellular proteins. Furthermore, the presence of five selected cellular proteins in the purified PRRSV virions was validated by Western blot and immunogold labeling assays.

## Results

### Purification of PRRSV virions

**Figure 1:**
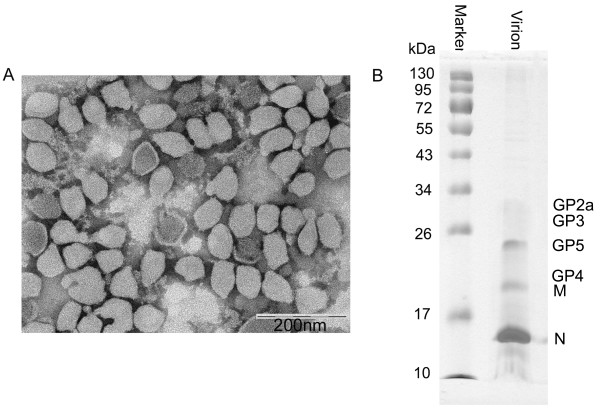
**Analysis of purified PRRSV virions**. (A) African green monkey kidney epithelial cell line (Marc-145) grown major particles PRRSV from CsCl coupled with sucrose density gradients purification, negatively stained with 3% potassium phosphotungstate, pH 6.5. (B) SDS-PAGE separation of proteins in a purified PRRSV preparation. 20 μg of proteins were separated on an polyacrylamide gel and stained with Coomassie blue.

Marc-145 cells were infected with a PRRSV strain i.e., GDBY1, isolated from dead pig[30]. 96 h post infection, the supernatant was harvested and concentrated through a 20% (w/v) sucrose cushion prepared in TNE buffer (Tris-buffered saline including 50 mM Tris, 100 mM NaCl, 1 mM EDTA, pH 7.4). For ultracentrifugation, the virion pellets were resuspended in TNE buffer and layered on the top of 10 to 50% CsCl gradient. There was a single faint opalescent band at 20-30% gradients. Subsequently, the opalescent PRRSV particles band was harvested and loaded onto 25-65% sucrose gradients. The higher density particles band in 35-45% soucrose gradient was collected and purified for a second time according to the same PRRSV purification method. The purity of virus preparation was directly examined by transmission electron microscopy following negative staining (Fig. 1). The PRRSV samples contained an abundance of virion particles without obvious contamination from host cellular material. For further identification of the virions protein composition, the purified virions were first separated by SDS-PAGE and then stained with Coomassie blue, three bright lanes (nucleocapsid protein N, membrane protein M and glycoprotein GP5) and three faint lanes (GP2a, GP3 and GP4) were found (Fig. 1). Some visible fainter bands that might represent cellular proteins incorporated into the virions were also observed. Taken together, the highly purified PRRSV particles were obtained.

The purity and quantity of PRRSV virions were crucial for proteomic analysis. Although porcine alveolar macrophages (PAM) are the main target cells of PRRSV, yet the infection by PRRSV of PAM primary cultures gave poor yields, making them impractical to obtain highly purified PRRSV virions for proteomic analysis. For another reason, the porcine genome is not yet fully annotated and this would restrict the identification of host proteins. Alternatively, the cell lines would support high levels of PRRSV growth and cells can be used to search the most extensive protein database (i.e. human).

### Proteomic analysis of PRRSV virions

**Figure 2:**
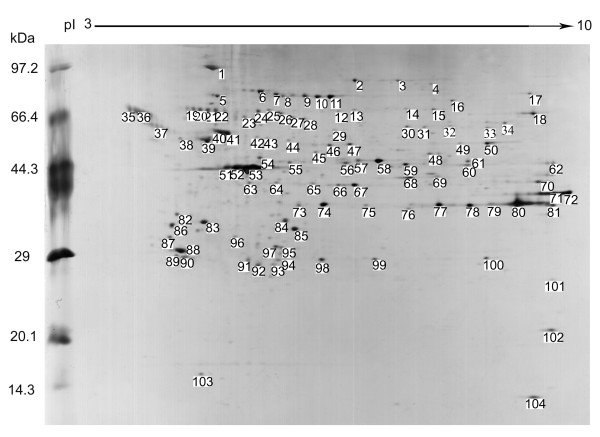
**Representative 2-DE gel images of purified PRRSV virions**. Protein (200 μg) was separated on the first dimensional pI 3-10 non linear IPG gels and second dimensional 5-17.5% continuous gradient vertical gels. The relative molecular mass is given on the right, while the isoelectric point is given on the top. Spots were analyzed by MALDI-TOF/TOF MS. The identified spots are numbered according to Table 2.

To obtain a detailed composition of PRRSV virion proteins, the highly purified virions were analyzed by 2-DE with 200 μg of protein loaded on 18 cm gel strip (pI 3-10). To minimize inter-gel and inter-sample variation, three repeats of independent sample preparations and three repeats of independent 2-DE PAGE were performed under identical conditions. All the gels provided high resolution spots for the separation of proteins. After image analysis, a total of 104 protein spots were detected on the silver stained gel (Fig. 2).

### Identification and functional classification of PRRSV-associated proteins

For finding the the identity of the 104 protein spots in 2-DE gel, all spots were excised, subjected to in-gel trypsin digestion and subsequent MALDI-TOF-TOF identification. The acquired MS/MS spectra were automatically searched against the nonredundant PRRSV proteins data base http://www.ncbi.nlm.nih.gov. Table 1 lists the predicted mass of each protein, the theoretical pI, the number of observed peptides and percent sequence coverage of the protein. Six structural proteins GP2a, GP3, GP4, GP5, M and N previously described to be in the PRRSV particles were successfully identified by one dimensional SDS-PAGE coupled with liquid chromatography tandem mass spectrometry (LC-MS/MS) liqumethod method (Table 1).

**Table 1:** Structural proteins of PRRSV identified by gel slice and LC-MS/MS.

Protein name	**Accession No**. ^**a**^	Protein score ion score	Protein MW(kDa)	**Protein pI **^**b**^	**Peptides Count **^**c**^	**Sequence coverage(%) **^**d**^
Glycoprotein 2a (GP2a)	gi|116006724	136	29.4	10.0	9	12.364

Glycoprotein 3 (GP3)	gi|52783626	112	29.01	8.36	7	9.721

Glycoprotein 4 (GP4)	gi|33307264	98	19.53	8.31	3	6.583

Glycoprotein 5 (GP5)	gi|7107031	108	22.41	9.40	9	11.278
Membrane protein	gi|10764662	120	19.03	9.99	13	14.325
Nucleocapsid protein	gi|61658266	149	13.51	10.4	16	34.278

a Accession numbers for NCBI database (accessible at http://www.ncbi.nlm.nih.gov/).

b Theoretical pI

c Observed peptides that differ either by sequence, modification or charge.

d Sequence coverge is based on peptides with unique sequence

The host proteins incorporated within PRRSV virions were analyzed on the basis of annotations from Uniprot Knowledge database (Swiss-Prof/TrEMBL) and Gene Ontology Databases. A total of sixty one host proteins were successfully identified and categorized into eight different groups as follows: cytoskeleton proteins, stress proteins, macromolecular biosynthesis proteins, metabolism-associated proteins, calcium-dependent membrane-binding proteins, glycoprotein, regualte apoptosis protein and other functional proteins (Table 2). The proteins are mostly host cellular cytoplamsic proteins, including cytosol, cytoskeleton, and cell organelles (e.g. Intermediate filament, microtube). In addition, some proteins are located in nucleus and membrane.

**Table 2:** Cellular proteins identified in purified PRRSV virions by 2-DE PAGE and MALDI-TOF/TOF MS

**Spot no**. ^**a**^	**Protein name **^**b**^	**Accession no**. ^**c**^	**Protein Score **^**d**^	**Protein MW(kDa) **^**e**^	**Protein pI **^**f**^	**Pep Count **^**g**^	**Intensity Matched **^**h**^	**Subcellular Location **^**i**^
**Cytoskeletal Proteins**								
7	keratin 10	gi|21961605	124/60	58.79	5.09	24	22.125	INF
12	coronin, actin binding protein, 1B	gi|197100107	414/338	55.68	5.96	18	38.007	C, CYS
27,29,40	keratin 9	gi|55956899	150/97	62.03	5.14	14	18.608	INF
69,101								
39	tubulin, beta polypeptide	gi|57209813	821/618	47.74	4.7	27	77.274	MIT, C
41	alpha-tubulin	gi|37492	679/534	50.13	5.02	23	72.834	MIT
42,43	tubulin, alpha, ubiquitous	gi|77539752	349/236	50.10	4.98	19	37.589	MIT
51,52,95	Beta-actin	gi|40744574	604/482	41.71	5.37	24	76.044	C, CYS
53	actin, gamma 1 propeptide	gi|4501887	797/602	41.77	5.31	26	77.03	C, CYS
58	keratin 1	gi|11935049	537/472	66.03	8.16	18	12.481	INF
82	tropomyosin 1 alpha chain isoform 4	gi|63252900	493/295	32.86	4.72	26	55.547	C, CYS
102	cofilin 1 (non-muscle)	gi|5031635	269/197	18.49	8.22	10	29.949	N, C, CYS
**Stress proteins**								
6	Heat shock 70 kDa protein 8 isoform 1	gi|5729877	938/675	70.85	5.37	38	72.617	C
24	Heat shock 60 kDa protein 1	gi|31542947	521/364	61.02	5.7	28	53.064	C
75	ribosomal protein P0	gi|4506667	572/462	34.25	5.71	14	41.767	C
94,98,99	Heat shock protein 27	gi|662841	449/339	22.31	7.83	14	57.414	C, N
**Metabolism-associated proteins**								
17	transketolase	gi|388891	495/382	67.84	7.89	16	57.92	CYO
18	pyruvate kinase	gi|35505	806/486	57.84	7.58	41	69.771	C, CYO
32	phosphoglycerate dehydrogenase	gi|23308577	526/397	56.61	6.29	23	42.346	C
33	aldehyde dehydrogenase 1A1	gi|21361176	601/461	54.83	6.3	22	51.887	C, CYO
34	UDP-glucose dehydrogenase	gi|4507813	600/350	54.99	6.73	33	72.634	C
49,50	enolase 1	gi|4503571	813/609	47.14	7.01	28	49.053	C
62	phosphoglycerate kinase 1A isoform 2	gi|4505763	738/553	44.59	8.3	26	53.6	C
71,72	glyceraldehyde-3-phosphate dehydrogenase	gi|37730278	654/543	23.85	9.17	13	38.336	C
73,84	guanine nucleotide binding protein (G protein), beta polypeptide 1	gi|197100735	253/176	37.35	5.47	15	27.248	ISPM
74	L-lactate dehydrogenase B	gi|4557032	493/335	36.62	5.71	18	64.612	C
78	Chain A, Fidarestat Bound To Human Aldose Reductase	gi|13096112	194/122	35.70	6.56	12	24.868	C
81	PREDICTED: lactate dehydrogenase	gi|109107094	457/283	36.61	8.45	24	56.639	C
96	peroxiredoxin 1	gi|4505591	433/305	22.10	8.27	16	54.055	C
97	proteasome activator hPA28 suunit beta	gi|1008915	271/191	27.33	5.44	13	29.728	CYO
100	triosephosphate isomerase 1	gi|4507645	677/547	26.65	6.45	17	54.228	CYO
**Macromolecular biosynthesis**								
14	chaperonin containing TCP1, subunit 3 (gamma)	gi|14124984	356/221	60.36	6.1	20	43.055	C
15	chaperonin containing TCP1, subunit 6A (zeta 1)	gi|197099952	541/389	58.04	6.3	23	55.446	C
26	chaperonin containing TCP1, subunit 5 (epsilon) protein	gi|24307939	700/424	59.63	5.45	40	64.77	C, N
30	chaperonin containing TCP1, subunit 2	gi|5453603	799/500	57.45	6.01	38	64.220	C, CYO
31	PRP19/PSO4 pre-mRNA processing factor 19 homolog	gi|7657381	425/304	55.15	6.14	21	31.179	N
37	retinoblastoma binding protein 4 isoform a	gi|5032027	277/202	47.63	4.74	13	41.617	N
54	eukaryotic translation initiation factor 4A isoform 1	gi|4503529	731/530	46.12	5.32	29	64.901	CYO
86	proliferating cell nuclear antigen	gi|49456555	513/400	28.69	4.57	16	36.423	N
**Glycoprotein**								
35,36	alpha2-HS glycoprotein	gi|2521981	73/51	35.64	5.2	10	10.222	S
**Calcium-regulated membrane-binding protein**								
79,80	Annexin A2	gi|18645167	769/568	38.55	7.57	28	83.608	S, EXS
83	Annexin A5	gi|4502107	234/122	35.91	4.94	13	39.159	C
85	Annexin A4	gi|4502105	640/465	36.06	5.84	26	78.847	C
104	S100 calcium binding protein A10	gi|4506761	275/210	11.20	6.82	6	23.65	MIT
**Regulate apoptosis**								
103	galectin-1	gi|4504981	257/236	14.7	5.25	12	20.78	C, S
**Others**								
13	T-complex protein 1 isoform a	gi|57863257	602/396	60.31	5.8	31	55.471	M
64,70	gastric-associated differentially-expressed proteinYA61P	gi|6970062	305/262	14.86	6.84	7	13.425	?

a Protein spot numbers on 2-DE gel.

b Alternative names are provided in parentheses.

c Accession numbers ford NCBI database (accessible at http://www.ncbi.nlm.nih.gov/).

d MASCOT protein score (based on combined MS and MS/MS spectra) of greater than 64 (p≤0.05) or the total ion score (based on MS/MS spectra) of greater than 30 (p≤0.05)

e Theoretical molecular mass.

f Theoretical pI.

g Observed peptides that differ either by sequence, modification or charge.

h Sequence coverge is based on peptides with unique sequence.

i The proteins subcellular location. INF-Intermediate filament; C-Cytoplasm; CYS-Cytoskeleton; N-Nucleus; CYO-Cytosol; ISPM-Internal side of plasma membrane;S- Secreted; EXS- Extracellular space; MIT- Mitochondrion; M- Membrane; ?-unknown

### Validation of cellular proteins by Western blot

**Figure 3:**
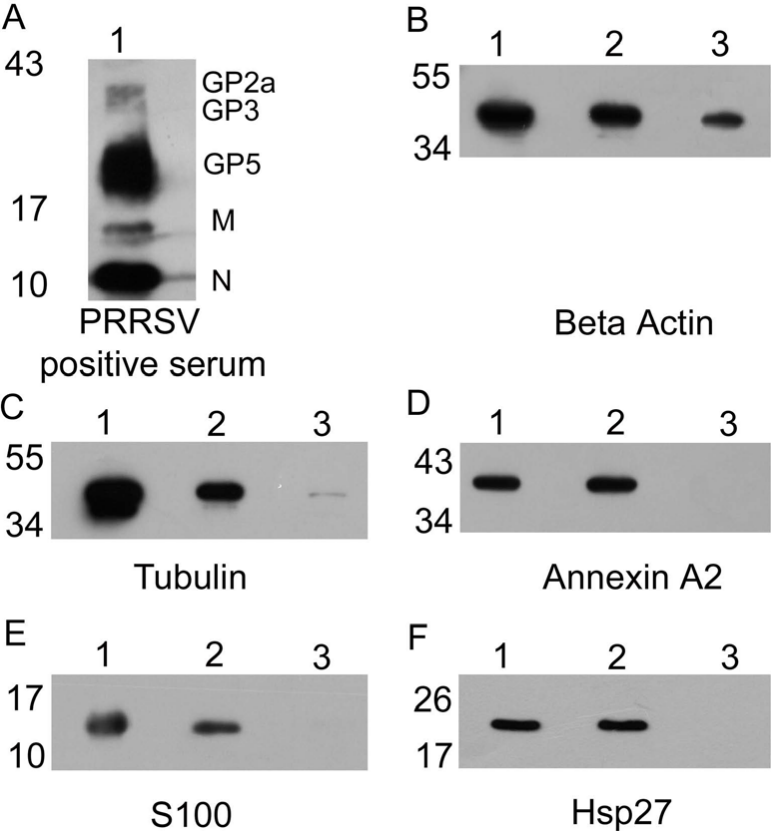
**Confirmation of virion-associated proteins by Western blot**. 10 μg of purified PRRSV virions (lane 1), protease overnight-digestion PRRSV virions followed by concentration through a sucrose cushion (lane 2) and purified Marc-145 cells lysate (lane 3) were analysed by Western blot. A, B, C, D, E and F indicate the immunoblot results using PRRSV positive serum, anti-beta Actin, anti-Tubulin, anti-Annexin A2, anti-S100, and anti-Hsp27 antibodies, respectively.

Following the identification of cellular proteins by proteomic method, immunoblot analysis was carried out to confirm their presence. In Western blot analysis, apart from five structural proteins, Beta actin, Tubulin, Annexin A2, S100 and Hsp27 were successfully detected both in purified PRRSV virions and protease-treated PRRSV virions (Fig. 3). In this study, the critical challenge was to prove that the host proteins were really an integral part of the virions and are not just non-specifically attached to the outside of the virions or derived from the contaminants. To address this question, the negative control of non-PRRSV infected Marc-145 cells lysates were purified using the same method as described earlier for PRRSV virions. Equal amounts of purified PRRS virions, protease-treated PRRSV virions purified Marc-145 cell lysates were included for Western blot analysis. Five structural proteins (GP2a, GP3, GP5, M and N) were identified in highly purified PRRSV virions. As shown in Fig 3, Beta actin, Tubulin, Annexin A2, S100 and Hsp27 were detected in purified virions and protease-treated virions. In addition, we detected no Annexin A2, S100 and Hsp27 in the negative control. Otherwise, it is an expectable result that we detected actin and tubulin in the non-viral infected MARC-145 cells lysate which might because of their high concentrations in all cells and subcellular fractions. The results revealed that the selected proteins are specifically packaged into PRRSV virions rather than contaminated proteins.

### Validation of cellular proteins by electron microscopy and immunogold labeling

**Figure 4:**
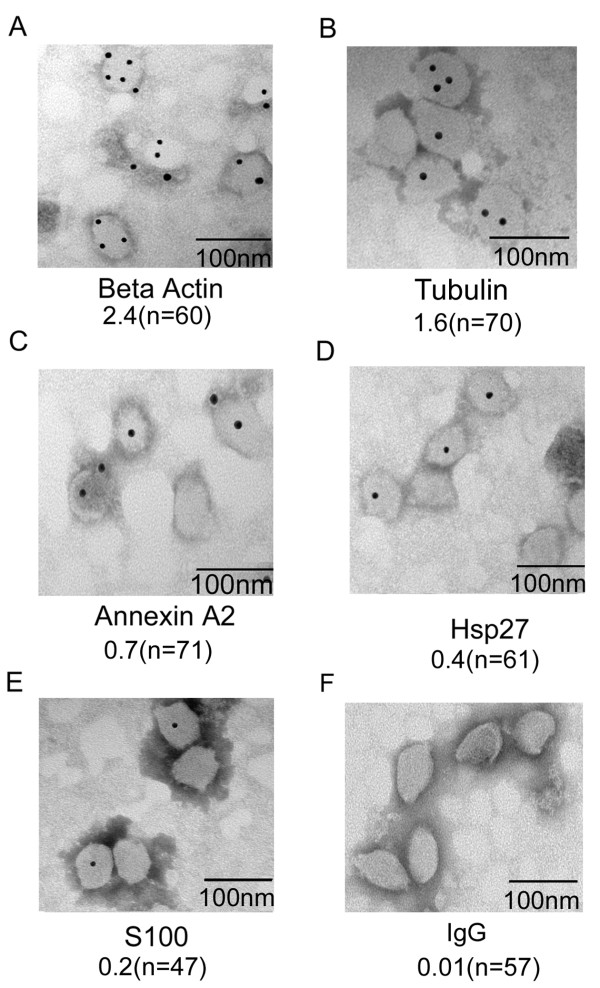
**Immunoelectron microscopy analysis of host proteins in purified PRRSV virions**. High purified PRRSV virions were immunogold labeled with antibodies against (A) Beta Actin, (B) Tubulin, (C) Annexin A2, (D) Hsp27, (E) S100 and (F) normal mouse IgG. The labeled virus were then negatively stained by phosphotungstic acid and examined under electron microscope (25,000 × magnification).The number of gold particles per virion is shown below (n = the number of virions counted).

In order to exclude the possibility of contaminants derived from inefficiently removed protease treatment, immuno-gold labling was performed for purified PRRSV, which provided additional evidence for the location of host cellular proteins in PRRSV virions. The subtilisin protease treated PRRSV virions were incubated with 1% v/v Triton X-100 for 2 min to increase the permeability of PRRSV envelope. By doing so, the microvesicles become lighter than the virions and virions can be isolated by density centrifugation. Proteins present inside the virion are protected by lipid envelope and therefore will be present after the protease treatment. Virus particles were incubated with antibodies of Beta Actin, Tubulin, Annexin A2, S100, Hsp27 and normal mouse IgG (Fig. 4) which were later on developed with a gold-conjugated secondary antibody. Binding of gold particles to PRRSV was then observed which showed the presence of many gold particles located on the surface of PRRSV virion for Beta actin, Tubulin and Annexin A2, meanwhile one or two gold particles on virion surface could be seen for S100 and Hsp27. However, almost no gold particles were established in PRRSV virions which were incubated with normal mouse IgG. The results indicated that the numbers gold particles were consistently related with proteins abundance in gel. Taken together, immunogold electron microscopy and Western blot data indicated that Beta actin, Tubulin, Annexin A2, S100 and Hsp27 were specifically incorporated into PRRSV virions.

## Discussion

PRRS is pandemic in swine producing regions throughout the world resulting in severe economic losses. However, the underlying mechanism of PRRSV pathogenesis remains to be well defined. In this study, we obtained highly purified PRRSV virions by CsCl gradients combined with sucrose gradients ultracentrifugation. Virion-associated proteins were identified by using 2-DE/MS proteomics approach followed by Western blot and electron microscopy. A total of six viral proteins and sixty one host proteins were successfully identified. Number of evidences show that some of virion-associated host proteins may play an important role in virus infectivity [31,32]. However, the relevance of cellular proteins to viral biology remains to be elucidated. This study provides strong evidence that cellular proteins are incorporated into enveloped viruses.

Three of structural proteins GP2a (pI 10.0), N (pI 10.4) and M (pI 9.99) all belong to alkaline proteins, therefore none of them could be detected by utilizing 2-DE gels. Several glycosylation sites have been identified on GP5 protein, which may change GP5 pI. Meanwhile, GP3 and GP4 which belong to non-major structural proteins were not identified on the 2-DE gels, which can be due to low concentration. Moreover, the two proteins (GP3 and GP4) which are involved in post-translational modification of glycosylation, and can change the proteins pI were not detected in 2-D gels. Our viral proteomics study of PRRSV virons identified several host cytoskeleton system proteins, which have the maximum profusion among the identified cellular proteins, including Actin, Keratin, Annexin, Coronin, Tubulin, Tropomyosin, and Cofilin. Enveloped viruses acquire their envelope through budding from the host cell, thus cytoskeletal proteins may be integrated inside the virions because of their propinquity to viral assembly and budding sites.

Available evidences indicate that host cytoskeletons, especially Actin, are involved in several animal virus budding processes [31]. Interestingly, actin was originally thought as a cellular contaminant, but later demonstrated to be an internal component of the measles virus [33,34]. The functional implication for assimilation of actin into these virus particles remains ambiguous. Actin has been suggested to play a role in the transport of synthetic viral RNA, which is presumably a prerequisite for budding [35]. In number of viruses, such as HIV and moloney murine leukemia virus actins are used to be very important during their budding [36-38]. Furthermore, actin and myosin form a dipolymer which play an important role in HIV 1 budding from host [39]. For influenza virus, actin plays indispensable roles during the endocytosis of the virus into polarized epithelia [40]. Beta-actin has been identified to interact with infectious bronchitis virus membrane protein and may play an important role in virion assembly and budding [29]. Keratin network is important for the intercellular transmission of persistent lymphocytic choriomeningitis virus infection and facilitates its own intercellular spread through the interaction between the viral nucleoprotein, keratin 1 and stimulation of cell-cell contacts [41]. Cytoskeletal filaments vimentin, cytokeratin 8, cytokeratin 18, actin, hair type II basic keratin and PRRSV receptor mediate the transportation of the virus in the cytosol [42]. Therefore, host cytoskeletion actin may play essential role in PRRSV budding or assembly.

In this study three annexin family members (A2, A4 and A5) were successfully identified in purified PRRSV virions. Annexin A2 is a calcium-regulated membrane-binding protein whose affinity for calcium is greatly enhanced by anionic phospholipids and implicated in a number of membrane-related events, including regulated exocytosis [43]. It binds calcium ions and may be involved in heat-stress response. Cellular annexin A2 was found to be endogenously associated with HCMV, HIV, influenza virus particles, and herpes simplex virus 1 [18,20,44,45]. HCMV-associated annexin A2 contributes to cell penetration by the virus, and is acquired during virus egress from infected cells and is bound to anionic phospholipids expressed on the virus surface, which may contribute to membrane binding and fusion events required for virus entry [44]. Annexin A2 as a cellular cofactor interactes with phosphatidylserine that eventually promotes HIV entry into MDM [46]. It is also demonstrated that annexin A2 interacts with HIV Gag at the phosphatidylinositol bisphosphate-containing lipid raft membrane domains, at which Gag mediates viral proper assembly in monocyte-derived macrophages, moreover ectopic expression of Annexin A2 in 293T cells increases HIV-1 production [47,48].

Our study showed that three S100 calcium binding proteins (S100 A6, S100 A10 and S100 A11) were also successfully identified in PRRSV virons. The calcium-dependent phospholipid-binding protein family members play a vital role in the regulation of cellular growth and signal transduction pathways. S100 calcium binding protein with 2 EF-hand calcium-binding motifs may function in stimulation of Ca^2+^-dependent insulin release, stimulation of prolactin secretion, and exocytosis. Hepatitis B virus (HBV) interacting with S100 A10 (p11) which binding to annexin II, have an important role in modulation of HBV function and implicates PML nuclear bodies and intracellular Ca^2+ ^in viral replication [49]. S100 and A2 form a heterotetramer and help in exocytosis [50].

Heat shock proteins may be potentially involved in all phases of the viral life cycle including cell entry, virion disassembly, viral genome transcription, replication and morphogenesis. Hsp27, Hsp60 and Hsp70 were also detected in this study. The heat-shock response activation might be a specific virus function ensuring proper synthesis of viral proteins and virions, thus stress proteins may also be important for virus replication [51]. Hsp27 was also reported in HIV and influenza virus [20,52], which was identified as a protein that specifically co-immunoprecipitates with HCV non-structural protein 5A and may be involved in HCV replication [53]. Hsp27 phosphorylation has been linked to an inhibition of NF-κB activation, suggesting that Hsp27 plays a role in HIV-1 infection of macrophages [54]. Moreover Hsp27 was observed to be up-regulated in PRRSV-infected PAMs [55]. Furthermore, Hsp90 complex which is incorporated into nucleocapsids are required for Hepadnavirus assembly and reverse transcription [56]. Purified primate lentiviral virions contain Hsp70 [52]. Some stress proteins, such as Hsp70 and Hsp90 are components for purified EBV virions and may regulate actin filament formation [28]. A proteomic analysis performed on highly purified HCV virions identified the heat shock cognate protein 70 (HSC70) as a part of the viral particles demonstrating that HSC70 modulates HCV infectivity and lipid droplet-dependent virus release [57].

Meanwhile, some metabolism-associated and macromolecular biosynthesis proteins were identified in PRRSV virions. Among these proteins such as Glyceraldehyde-3-phosphate dehydrogenase, Enolase 1, Phosphoglycerate dehydrogenase, Pyruvate kinase were also demonstrated in SARS, Influenza virus, HIV-1 and rhesus monkey rhadinovirus [18,20,21,58]. However, functions of these proteins in virus life cycle have not been well understood. Eukaryotic translation initiation factors are ATP-dependent RNA helicase which form an exon junction complex (EJC) in splicing exon-exon junctions of mRNA. Influenza virus NS1 protein recruits eIF4GI specifically to the 5' untranslated region (5' UTR) and act as a translational activator for virus mRNA [59,60]. Eukaryotic translation initiation factor 4A detected in PRRS virions may bind to virus replicase and form a transcription complex which may play a key role in PRRSV transcription and genome replication.

According to our investigation, guanine nucleotide-binding proteins, tyrosine 3-monooxygenase/tryptophan 5-monooxygenase, peroxiredoxin 1 and galectin-1 protein were first reported in PRRSV compared to other enveloped virus. Heterotrimeric guanine nucleotide-binding proteins integrate signals between receptors and effector proteins. Tyrosine 3-monooxygenase/tryptophan 5-monooxygenase activation protein interacts with IRS1 protein, suggesting a role in regulating insulin sensitivity. In response to DNA damage, proliferating cell nuclear antigen protein is ubiquitinated and involved in the RAD6-dependent DNA repair pathway. Peroxiredoxin 1 protein may play an antioxidant protective role in cells and contribute to the antiviral activity of CD8^+ ^T cells. Galectin-1, a dimeric beta-galactoside-binding protein which acts as a soluble adhesion molecule by facilitating attachment of HIV-1 to the cell surface and facilitates HIV-1 infection by promoting early events of the virus replication cycle (i.e. adsorption) [61,62].

## Conclusions

Apart from six structural proteins, we successfully identified sixty one virion-associated host cell proteins in purified PRRSV virions by 2-DE coupled with MALDI-TOF/TOF MS proteomic approach. In addition, we utilized Western blot and immuno-gold labeling assays to verify the presence of cellular proteins and determine their location inside the PRRSV virions. Taken together, the selected proteins i.e., Actin, Tubulin, Annexin A2, S100 and Hsp27 were demonstrated to incorporate into PRRSV virions. We contemplate that most of host proteins identified are enclosed into the virion particles and may be functionally involved in PRRSV life cycle, pathogenesis and virulence. Moreover, the identification of cellular proteins in PRRSV virions has great practical implications, providing potential targets for novel approaches to control PRRSV infection.

## Methods

### Propagation and purification of PRRSV

African green monkey kidney epithelial cell line (Marc-145) is a very convenient research model as PRRSV host cell. Marc-145 cells with 80% confluence were infected with high pathogenic PRRSV grouped into Type II (Genebank accession no: GQ374442) at a multiplicity of infection (MOI) of 0.01. After 72-96 h post infection, the supernatant was harvested and clarified by centrifugation at 10,000 × *g *at 4°C for 30 min (Eppendorf 5804 R). PRRSV particles were concentrated by ultracentrifugation through a 20%(w/v) sucrose cushion prepared in TNE buffer [50 mM Tris-HCl (pH 7.5), 100 mM NaCl, 1 mM EDTA]. The virus pellet was resuspended in TNE buffer, layered on the top of 10-50% (w/v) CsCl gradients and concurrently centrifuged at 160,000 × *g *(SW 40 rotor, Beckman) at 4°C for 12 h. The banded virus was collected, diluted with TNE buffer and then layered on the top of 25-65% (w/v) sucrose gradients and at the same time centrifuged at 160,000 × *g *(SW 40 rotor, Beckman) at 4°C for 4 h. The PRRSV particles band were harvested and pelleted at 160,000 × *g *for 2 h to remove the traces of sucrose. In order to get highly purified PRRSV virions, the collected banded virus was purified for a second time according to the same purification procedure. The purified virus was stored at -80°C for further use.

### Validation of purified PRRSV virions by electron microscope and SDS-PAGE

Highly purified virus (3 μl) was adsorbed to Formavar-supported, carbon-coated nickel grids (230 mesh) for 2 min at room temperature (RT). The grids were then negatively stained with 3% phosphotungstic acid and examined under a JEM-1400 electron microscope (JEM-100CX-II, JEOLLTD, Japan) operated at 120 kV.

SDS-PAGE was performed to validate the purified PRRSV virions. Proteins from the purified virus (20 μg) were denatured at 100°C for 10 min in 1 × (SDS-PAGE) sample buffer and were then separated by SDS-PAGE. Coomassie Blue R250 was used for protein staining.

### Two-dimensional (2-DE) separation of proteins of purified PRRSV virions

The highly purified PRRSV virions were lysed in lysis buffer (7 M urea, 2 M thiourea, 2% Triton X-100, 100 mM DTT, 0.2% IPG buffer, pI 3-10) containing protease inhibitor cocktail (Sigma) for 1 h at 4°C. After lysing by sonication for 5 min with 40% power output, the lysates were clarified by centrifugation at 20,000 × *g *for 20 min at 4°C. The supernatant was collected and the concentration was determined by 2-DE Quant kit (Amersham, USA).

The first-dimension separation was performed using 18 cm immobilized pH gradients (IPG) strips at nonlinear pI 3-10 (GE Healthcare) for isoelctric focusing (IEF) and vertical SDS-PAGE for second dimension. The IPG strips were rehydrated with 350 μl of rehydration buffer (7 M urea, 2 M thiourea, 2% CHAPS, 65 mM DTT, 0.5% IPG buffer pI 3-10 NL) containing 200 μg protein for 12 h at 20°C with passive rehydration. IEF was performed as follows:100 V, linear, 200 Volt-Hours (Vhs); 200 V, gradient, 200 Vhs, 500 V, linear, 500 Vhs; 1,000 V, linear, 2,000 Vhs; 4,000 V, gradient, 4,000 Vhs; 8,000 V, linear, 32,000 Vhs. The IPG strips were equilibrated for 15 min with gentle shaking in equilibration buffer (6 M urea, 30% glycerol, 2% SDS, 0.375 M Tris-HCl, pH 8.8) containing 2% DTT, followed by additional equilibration for 15 min in SDS equilibration buffer containing 2.5% iodoacetamide. The second-dimensional separation was carried out by using 5-17.5% continuous gradient SDS-PAGE. The gels were stained by the modified silver staining method compatible with MS and scanned at a resolution of 600 dpi using ImageScanner(Amersham Pharmacia Biotech). The image analysis was carried out with Image Master 2D Platinum 5.0 according to the manufacture's protocol (GE Healthcare).

### In-gel tryptic digestion

The protein spots on the silver-stained gels were excised and transferred into 0.5 ml Eppendorf tubes, washed 3 times with ddH_2_O, destained in 1:1 solution of 30 mM potassium ferricyanide (K_3_Fe(CN)_6_) and 100 mM ammonium bicarbonate (NH_4_HCO_3_). After hydrating with 100% acetonitrile (ACN) and drying in a SpeedVac for 20 min, the gels were rehydrated in a minimal volume of sequencing grade porcine trypsin (Promega, USA) solution (20 μg/ml in 25 mM NH_4_HCO_3_) and incubated at 37°C overnight. The supernatant was collected and transferred into a 200 μl microcentrifuge tube, while the gels were extracted once with extraction buffer (67% ACN containing 5% trifluoroacetic acid TFA) at 37°C for 1 h. Finally, the peptide extracts and the supernatant of the gel spots were combined and then completely dried in a SpeedVac centrifuge.

### MALDI-TOF/TOF MS, MS/MS analysis and database search

Protein digestion extracts were resuspended with 5 μl of 0.1% TFA, and then the peptide samples were mixed (1:1 v/v) with a matrix consisting of a saturated solution of α-cyano-4-hydroxy-trans-cinnamic acid (CHCA) in 50% ACN containing 0.1% TFA. Digested protein (0.8 μl) of each sample was spotted onto stainless steel target plates and allowed to air-dry at room temperature. Three bright bands were cut from one dimensional polyacrylamide gel ranging from the molecular masses of 10-26 kDa, and subjected to *in situ *tryptic digestion.

Peptide mass spectra were obtained on an Applied Biosystem Sciex 4800 MALDI TOF/TOF Plus mass spectrometer (Applied Biosystems, Foster City, CA). Data were acquired in positive MS reflector using a CalMix5 standard to calibrate the instrument (ABI 4700 Calibration Mixture). Mass spectra were obtained from each sample spot by accumulation of 900 laser shots in a mass range of 800-3500. For MS/MS spectra, the 5-10 most abundant precursor ions per sample were selected for subsequent fragmentation and 1,200 laser shots were accumulated per precursor ion.

Combined MS and MS/MS spectra were submitted to MASCOT searching engine (Matrix Science, London, UK) by GPS Explorer software (Applied Biosystems) for proteins identification. Parameters for searches were as follows: taxonomy of primates, trypsin of the digestion enzyme, one missed cleavage site, partial modification of cysteine carboamidomethylated and methionine oxidized, none fixed modifications, MS tolerance of 60 ppm, MS/MS tolerance of 0.25 Da. A total of 133,518 sequences in the database actually were searched. MASCOT protein score (based on combined MS and MS/MS spectra) of greater than 64 (p≤0.05) or the total ion score (based on MS/MS spectra) of greater than 30 (p≤0.05) were accepted.

### PRRSV structural proteins identification from one dimensional SDS-PAGE

The visible proteins were cut from SDS-PAGE gel and subjected to *in situ *tryptic digestion prior to mass spectrometric analysis. EttanTM MDLC system (GE Healthcare) was applied for desalting and separation of tryptic peptides mixtures. In this system, samples were desalted on RP trap columns (Zorbax 300 SB C18, Agilent Technologies), and then separated on a RP column (150 μm i.d., 100 mm length, Column technology Inc., Fremont, CA). Mobile phase A (0.1% formic acid in HPLC-grade water) while the mobile phase B (0.1% formic acid in acetonitrile) were selected. 20 μg of tryptic peptide mixtures was loaded onto the columns, and separation was done at a flow rate of 2 μl/min by using a linear gradient of 4-50% B for 120 min. A Finnigan TM LTQTM linear ion trap MS (Thermo Electron) equipped with an electrospray interface was connected to the LC setup for eluted peptides detection. Data-dependent MS/MS spectra were obtained simultaneously. Each scan cycle consisted of one full MS scan in profile mode followed by five MS/MS scans in centroid mode with the following Dynamic Exclusion TM settings: repeat count 2, repeat duration 30 s, exclusion duration 90 s, while each sample was analyzed in triplicate.

MS/MS spectra were automatically searched against the non-redundant PRRSV protein data base http://www.ncbi.nlm.nih.gov. The peptides were constrained to be tryptic and up to two missed cleavages were allowed. Carbamidomethylation of cysteines were treated as a fixed modification, whereas oxidation of methionine residues was considered as variable modifications. The mass tolerance allowed for the precursor ions and fragment ions was 2.0 Da and 0.0 Da, respectively. The protein identification criteria were based on Delta CN (≥0.1) and cross-correlation scores (Xcorr, one charge≥1.9, two charges ≥ 2.2, three charges ≥ 3.75).

### Protease treatment of PRRS virions

Purified PRRSV particles equivalent to 50 μg protein was incubated with 100 μg subtilisin protease (Sigma) for 14 h at 37°C [20]. The treated virus was diluted to l ml in TNE buffer and added 10 μl Cocktail (Sigma). The treated virus particles were centrifuged through 25-65% sucrose gradients at 160,000 × *g *(SW 40 rotor, Beckman) at 4°C for 4 h. The PRRS virion were subjected to Western blot analysis after sedimentation.

### Validation of cellular proteins by Western blot

Mouse monoclonal antibodies against Actin, Heat shock protein Hsp27 and S100 were purchased from Millipore, and rabbit polyclonal antibody against Annexin A2 and Tubulin were products of Abcam Corporation. The negative control of non-viral infected MARC-145 cells lysate was prepared by using the same method as purifying PRRSV virions. Equal amounts of purified PRRS virions, protease-treated PRRSV virions and purified Marc-145 cells lysate were suspended in 1 × loading buffer (50 mM Tris-HCl pH 6.8, 2% SDS, 0.1% bromophenol blue, 10% glycerol, 100 mM DTT) and denatured by heating at 100°C for 5 min. After separated by SDS-PAGE, the viral proteins were transferred to ployvinylidene difluoride (PVDF) membrane (Millipore) for 20 min at 15 V. The membrane was then blocked in 5% nonfat milk-Tris buffered saline buffer (TBS)-0.1% Tween-20 overnight at 4°C. The PVDF membrane was washed three times with TBS plus 0.2% Tween-20 and incubated with properly diluted primary antibodies for 2 h at RT. Following three washes with TBS, the secondary antibody conjugated to horseradish peroxidase (HRP) was added for 1 h at RT. Immunoreactive protein bands were visualized with ECL plus Western Blot Detection System (Kodak, NY, USA).

### Validation of cellular proteins by electron microscopy and immunogold labeling

In order to assess the locations of the host proteins in the PRRSV particles, the immunoelectron microscopy technique was performed as previously described [63]. Aliquots (3 μl) of protease treated of PRRS virions were adsorbed on the grid and was thoroughly washed for 5 min in TBS buffer (50 mM Tris-Cl pH 7.5, 150 mM NaCl) placed on parafilm. For detergent treatments, after removing the excess fluid by touching the edge of grids with filter paper, the grids were then covered with 1% alkyl phenoxy polyethoxy ethanol (Triton X-100) for 2 min. The grids were then washed with distilled water and then blocked with 5% bovine serum albumin (BSA) in TBS for 45 min. Blocking reagent was removed, and grids were incubated on a drop of primary antibody solution (diluted 1:100 in BSA/TBS) for 1 h at RT. Following three times thorough wash with TBS, the grids were incubated with the secondary antibody goat anti-rabbit IgG conjugated with gold particles (6 nm in diameter, Abcam) for 1 h at RT. The unbound antibodies were removed, and the grids were thoroughly washed and negatively stained with 3% phosphotungstic acid (pH 6.5) for 30 s. Negatively stained virions were examined on a scan and transmission electron microscope.

## Abbreviations

PRRSV: porcine reproductive and respiratory syndrome virus; PAM: primary cultures of porcine alveolar macrophages; MALDI-TOF: matrix-assisted laser adsorption ionization-time of flight; LC-MS: liquid chromatography tandem mass spectrometry; 2-DE: two-dimensional gel electrophoresis; CSCL: cesium chloride; HRP: horseradish peroxidase; PI: isoelectric point; MW: molecular weight; RT: room temperature; MS: mass spectrometric; SDS-PAGE: sodium dodecyl sulfate-polyacrylamide gel electrophoresis; IEF: isoelctric focusing; BSA: bovine serum albumin; TBS: tris buffered saline; IPG: immobilized pH gradients; DTT: dithiothreitol; IAA: iodoacetamide; ACN: acetonitrile; TFA: trifluoroacetic acid.

## Competing interests

The authors declare that they have no competing interests.

## Authors' contributions

CZ performed the main proteomic experiments, data analysis and drafted the manuscript. CX participated in the detailed experimental design. YL and XL assisted in the propagation and purification of PRRSV. QK and XR contributed to the initial phase of the proteomic experiments. DS, YB and YC conceived study, and participated in its design, coordination and helped to sketch the manuscript. All authors have read and approved the final manuscript.
